# Recurrent Type A Aortic Dissection and New Entry after Endovascular Repair

**DOI:** 10.3400/avd.cr.26-00098

**Published:** 2026-09-26

**Authors:** Akihito Matsushita, Takashi Hattori, Wahei Mihara

**Affiliations:** 1 Department of Cardiovascular Surgery, Teikyo University Chiba Medical Center, Ichihara, Chiba, Japan; 2 Department of Cardiovascular Surgery, Seikeikai Chiba Medical Center, Chiba, Chiba, Japan

**Keywords:** aortic dissection, thoracic endovascular aortic repair (TEVAR), postoperative complication

## Abstract

Thoracic endovascular aortic repair (TEVAR) for acute type B aortic dissection can be complicated by retrograde type A aortic dissection (RTAD) and stent graft-induced new entry (SINE). A 77-year-old man developed acute type A aortic dissection after TEVAR and subsequently experienced recurrent RTAD and 3 episodes of distal SINE despite multiple surgical and endovascular interventions, eventually dying of postoperative bleeding. This case highlights the potential for repeated RTAD and SINE in the same patient and underscores the importance of careful surgical planning and endovascular device selection in patients with fragile, tortuous dissected aortas.

## Introduction

Thoracic endovascular aortic repair (TEVAR) has become the standard treatment for acute type B aortic dissection. However, retrograde type A aortic dissection (RTAD) and stent graft-induced new entry (SINE) remain serious complications after TEVAR.^1–5)^

## Case Report

A 77-year-old man with a history of type B aortic dissection presented with acute chest pain. He had undergone TEVAR using a 34-mm Conformable GORE TAG (W. L. Gore & Associates, Flagstaff, AZ, USA) and endovascular repair for an abdominal aortic aneurysm within the previous year at another hospital. Computed tomography (CT) showed acute type A aortic dissection with an entry in the ascending aorta, distant from the proximal edge of the stent graft (**Fig. 1**).

**Fig. 1:**
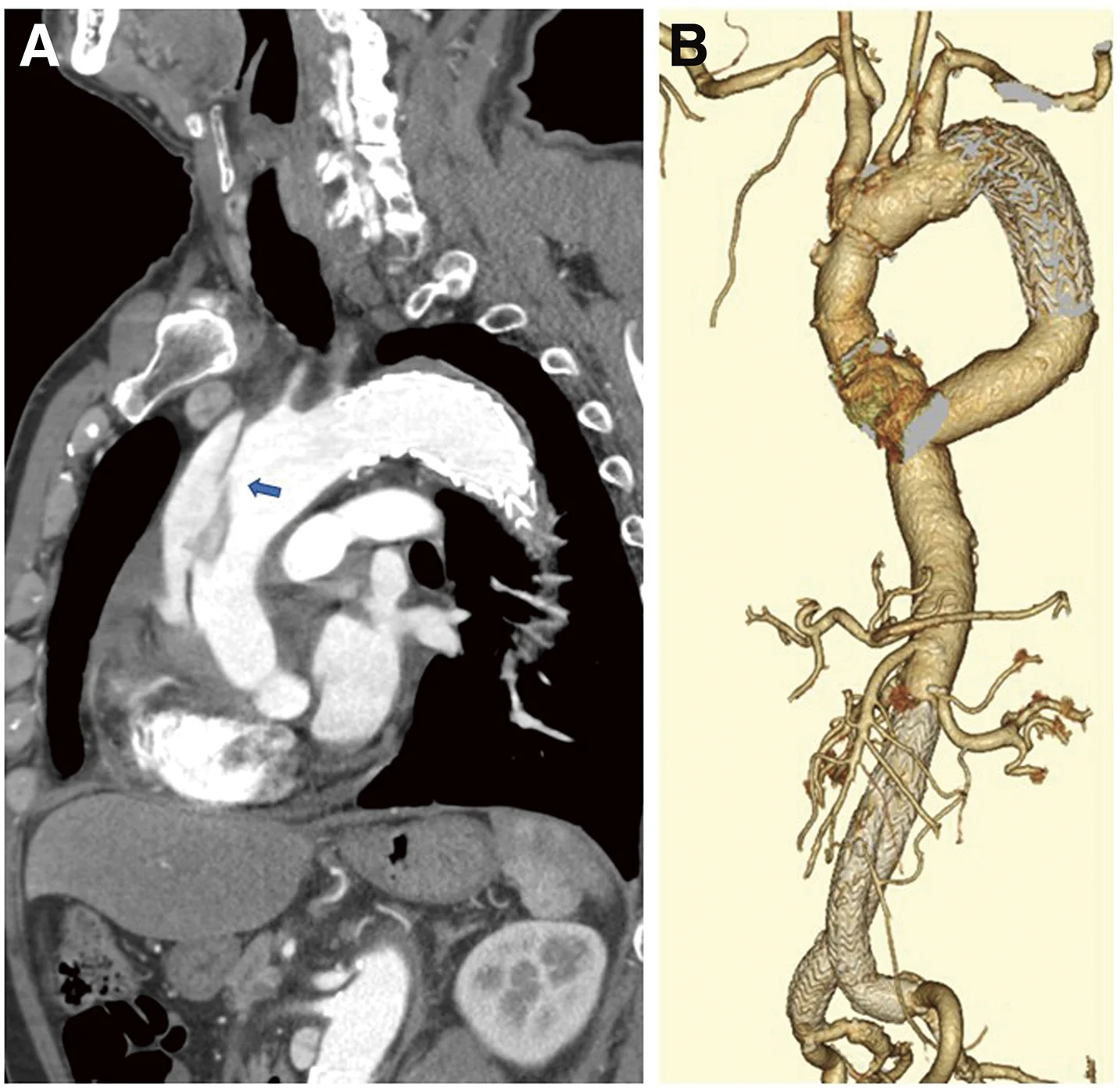
CT showing (**A**) acute type A aortic dissection with an entry in the ascending aorta (arrow). (**B**) CT images after ascending aortic graft replacement. CT, computed tomography

Emergency ascending aortic replacement using a 24-mm woven shield graft (J Graft SHIELD NEO; Japan Lifeline, Tokyo, Japan) was performed under circulatory arrest. Intraoperative findings confirmed no direct relationship between the entry and the proximal stent-graft edge. Pathological examination revealed no specific abnormality, and hereditary disease was excluded. The patient was discharged without complications on postoperative day 25.

Four months later, he returned with dyspnea and lower extremity edema. Echocardiography demonstrated an aortic pseudoaneurysm, pulmonary artery shunt flow, and severe pulmonary hypertension with a mean pulmonary artery pressure of 62 mmHg. CT showed pulmonary artery perforation and recurrent type A dissection with rupture at the aortic arch, distant from both the distal anastomosis and the proximal stent-graft edge (**Fig. 2**). Emergency total arch replacement using a 4-branched woven shield graft (J Graft SHIELD NEO) under circulatory arrest with selective cerebral perfusion was performed, along with direct closure of the pulmonary artery perforation. Postoperatively, mediastinitis caused by methicillin-resistant coagulase-negative staphylococci required sternal resection and omental flap transposition. He recovered and was discharged on postoperative day 47.

**Fig. 2:**
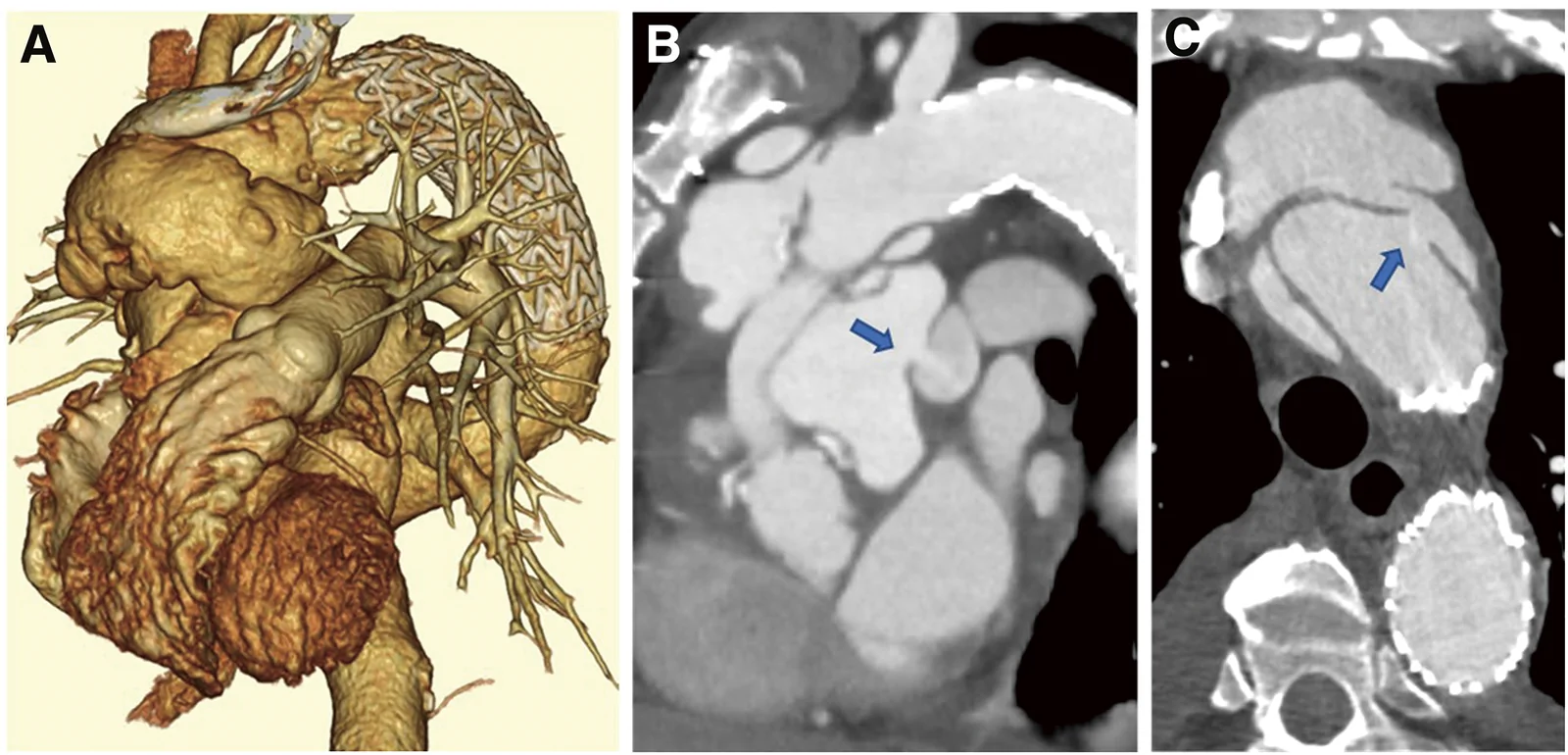
Computed tomography showing (**A**) recurrent type A aortic dissection at the aortic arch 4 months after the initial operation. (**B**) Pulmonary artery perforation was also confirmed at the same time (arrow). (**C**) Entry was located away from the initial anastomosis (arrow).

Two months later, he developed sudden back pain. CT demonstrated distal SINE at the distal end of the initial stent graft (**Fig. 3A**). The stent graft was fully expanded to its nominal diameter of 34 mm, whereas the distal landing zone measured 31 mm in diameter after complete resolution of the thrombus associated with the previous type B aortic dissection. Emergency additional TEVAR was performed for impending rupture using a 34-mm Conformable GORE TAG with active control, for which the manufacturer’s recommended aortic diameter range is 27–32 mm. The distal landing zone measured 31 mm in diameter, resulting in a stent graft-to-aorta diameter ratio of 109.7% (34/31), corresponding to 9.7% oversizing. Device delivery was difficult because the guidewire repeatedly entered the false lumen through the SINE, requiring a tug-of-wire pull-through technique between the brachial and femoral arteries. He recovered and was discharged 13 days later. CT performed before discharge demonstrated that the stent graft had expanded to 30 mm in diameter at its distal end (**Fig. 3B**).

**Fig. 3:**
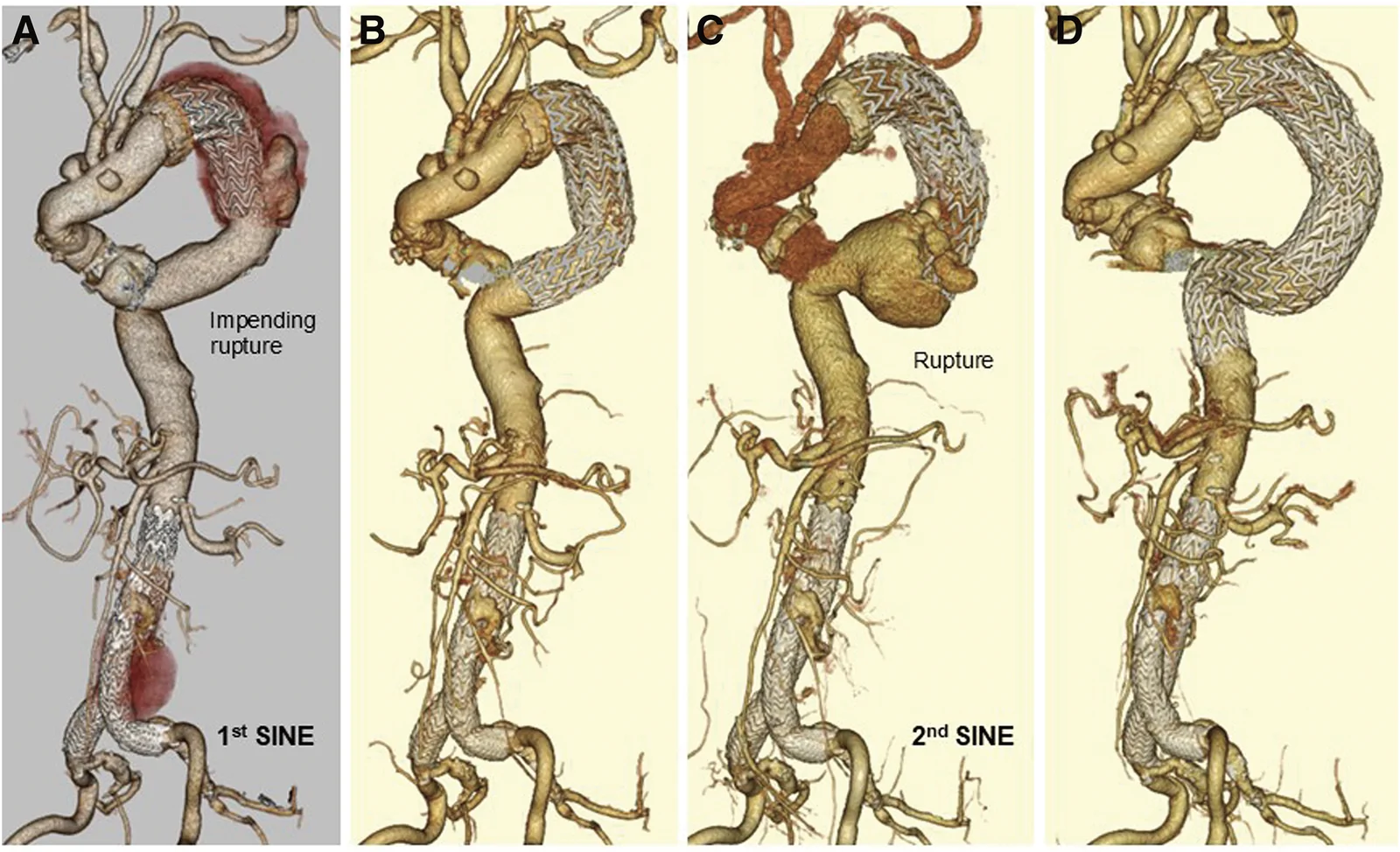
Serial computed tomography images demonstrating recurrent distal SINE after TEVAR. (**A**) First distal SINE at the distal end of the initial stent graft. (**B**) Additional TEVAR performed for the first distal SINE. (**C**) Second distal SINE with rupture and lung perforation distal to the additional stent graft. (**D**) Third TEVAR performed for recurrent distal SINE. SINE, stent graft-induced new entry; TEVAR, thoracic endovascular aortic repair

After another 2 months, he presented with hemoptysis and back pain. CT revealed recurrent distal SINE and lung perforation (**Fig. 3C**). As with the previous SINE, the stent graft was fully expanded to 34 mm at its distal end, while the distal landing zone remained 31 mm in diameter after complete resorption of the thrombus associated with the previous type B aortic dissection. Emergency third TEVAR using the same stent graft was performed successfully (**Fig. 3D**). Until the second distal SINE, elevated C-reactive protein levels were observed at the time of each vascular event; however, the organism identified on the initial occasion was not detected in subsequent blood cultures. After the third TEVAR, persistent inflammatory changes and periaortic air around the descending aorta remained despite prolonged antibiotic therapy, and blood cultures later grew *Haemophilus parainfluenzae*. CT performed 1 month after the third TEVAR, following a month of antibiotic therapy, demonstrated persistent air around the site of the omental flap transposition performed for mediastinitis following the previous TAR. A small amount of periaortic air around the descending aorta at the distal end of the stent graft, which had been present immediately after the procedure, also persisted. The stent-graft diameter at the distal end remained unchanged at 31 mm. Two months after the third TEVAR, recurrent back pain developed, and CT showed a third distal SINE with lung perforation distal to the prior repair. At the site of the SINE, the stent graft was fully expanded to its nominal diameter of 34 mm. Emergency descending aortic replacement using a 30-mm woven shield graft (J Graft SHIELD NEO) was performed, but the patient died from postoperative bleeding.

## Discussion

A recent meta-analysis reported RTAD after TEVAR in 2.5% of patients, with a mortality rate of 37.1%.^1)^ Acute dissection and underlying aortic dissection are important risk factors.^1)^ In the present case, the fragile dissected aorta and marked tortuosity may have contributed to repeated RTAD and SINE.

Previous reports have demonstrated that RTAD entries are frequently located away from the stent-graft edge.^2,3)^ Although ascending aorta replacement is often selected when the entry is confirmed to be in the ascending aorta, our experience suggests that total aortic arch replacement may be considered even when the entry is distant from the stent graft because recurrence can occur.

Distal SINE reportedly occurs in 4.8% of TEVAR patients, with recurrent SINE in approximately 5.0%.^4)^ Moreover, Czerny et al. reported that SINE occurs with similar incidence after acute or chronic dissection and with a higher degree of stent graft oversizing in patients who developed SINE.^4)^ Li et al. also reported that recurrence of SINE was high after re-TEVAR using routine stent grafts and found that more tapered stent grafts may be helpful in preventing the recurrence of SINE.^5)^ In our case, the selected device was within the recommended range for the target aortic diameter, and the distal oversizing was 9.7%, which was not excessive. Nevertheless, a more tapered device may have been preferable given the anatomical configuration. In addition, we should pay as much, or more, attention to additional TEVAR rather than initial TEVAR with respect to the technique, including the guidewire and delivery system, which can cause potential damage to the aortic wall. Aortic replacement with open left thoracotomy can be considered an option for additional treatment of SINE, mainly if the tug-of-wire method or balloon touch-up must be used for the tortuous nature of the aorta.

In this case, infected pseudoaneurysm was also considered an important differential diagnosis because the CT findings resembled graft infection with anastomotic dehiscence, and the history of mediastinitis may have contributed to tissue fragility. Although no pathological evidence suggesting giant cell arteritis or other large-vessel vasculitis was identified, these disorders cannot be completely excluded because inflammatory lesions may be segmental. However, considering the absence of clinical manifestations suggestive of vasculitis and the presence of persistent postoperative infection, infection-related tissue fragility was considered a more plausible explanation in the present case. Therefore, in addition to procedural factors, bacterial infection itself may also have played a role in pseudoaneurysm formation, although recurrent SINE was considered the predominant mechanism in this case.

## Conclusions

In summary, this case demonstrates repeated RTAD and recurrent distal SINE after TEVAR in the same patient. Careful selection of the operative strategy and endovascular device is essential in patients with a fragile and tortuous dissected aorta.
